# Dreaming during the COVID-19 pandemic: Support for the threat simulation function of dreams

**DOI:** 10.3389/fpsyg.2023.1124772

**Published:** 2023-02-06

**Authors:** Noor H. Abbas, David R. Samson

**Affiliations:** Sleep and Human Evolution Lab, Department of Anthropology, University of Toronto Mississauga, Mississauga, ON, Canada

**Keywords:** COVID-19, dreams, threat simulation theory, social simulation theory, dream recall, evolution

## Abstract

Evolutionary theories suggest that dreams function as a world simulator of events that maximizes our ability to surmount social and threat-related challenges critical to survivorship and reproduction. Here, in contrast to the incorporation continuity hypothesis, we test the (1) social bias hypothesis, which states that dreams will overrepresent positive social interactions relative to waking life, (2) the mutually exclusive threat bias hypothesis, the idea that dream content will be negative relative to waking life, (3) the strengthening hypothesis, which states that dreams will rehearse more positive interactions with individuals the self is familiar with relative to waking life, and (4) the compensation hypothesis, which states that social contents in dreams increases during periods of social seclusion. Dream (*n* = 168) and wake (*n* = 184) reports were collected through a standardized online survey from 24 undergraduate students. Recalls were analyzed using the Social Content Scale. Generalized linear mixed effects models were used, and the following fixed-effects were considered for the study; the number of reports contributed, report state, biological sex, stress, social support, and media exposures. Results showed support for the threat bias hypothesis, we found that dreams were more negative and featured more unfamiliar individuals in contrast to waking life. Additionally, we found partial support for the social bias and the strengthening hypotheses, however no support was shown for the compensation hypothesis. Overall, these results demonstrate support for the threat simulation function of dreams.

## 1. Introduction

Humans spend a significant portion of nighttime sleep dreaming. Historical research suggests that dreams have been a topic of interest since the dawn of recorded history (Freud, 1900; Hughes,2000; Zhang and Guo, 2018). The history of modern dream research originates with Sigmund Freud and Carl Jung, who both essentially claimed that dreams are metaphorical expressions of unconscious desires (Freud, 1900; Graveline and Wamsley, 2015). In contrast to this is Alan Hobson’s *activation synthesis model*, which essentially claims that dreams are random biproducts of rapid eye-movement (REM) sleep physiology (Hobson and McCarley, 1977; Foulkes, 1985; Hobson, 2009; Graveline and Wamsley, 2015). This model was later refined into modern *dream continuity theory*, which claims that dreams, broadly construed, reflect waking concerns and assumes a mirroring of waking perceptions, concerns, and contents within dreams (Hall and Nordby, 1972; Schredl and Hofmaan, 2003; Domhoff and Schneider, 2018; Schredl, 2018). Specifically, the ***incorporation continuity hypothesis**,* is often treated as the null hypothesis in dream research. It assumes that no specific contents are biased to appear in either sleep or wake states and dreams mirror waking life preoccupations (Revonsuo et al., 2015a,b).

Evolutionary theories regarding dream functions have proliferated, which posit that dreams are either remarkably social phenomenon or are inherently threatening in nature—both being ecological conditions that humans have presumably been surrounded by in Paleolithic ancestral environments (Salmon and Crawford, 2008; Sutcliffe et al., 2012). Research demonstrates that dreams meet the three necessary criteria for natural selection: genetic variation, inheritance, and differential fitness (Franklin and Zyphur, 2005). It has been suggested that dreams demonstrate a preparatory function that ultimately served to increase fitness of the self (Revonsuo, 2000; Tuominen et al., 2019), thus being subject to natural selection. Dependent on the ecological conditions the self is in, the functions of dreams are to serve either one of two purposes: by way of mental rehearsal, dreams essentially prepare the self to respond to threats and/or social situations, with each complementary theory termed as threat simulation and social simulation, respectively (Revonsuo, 2000; Dresler, 2015; Revonsuo et al., 2015a,b; Tuominen et al., 2019; Sterpenich et al., 2020). This *simulation theory of dreaming,* is a framework that broadly considers dreams as serving the function of world-simulation constructs. Specifically, dreaming functions as episodic simulations that are credible real-world analogs (Domhoff and Schneider, 1996; Hobson, 2009; Dresler, 2015; Revonsuo et al., 2015a,b; Tuominen et al., 2019) that can rehearse and prepare the individual for waking life, which offers a preparatory advantage to the self if a similar situation is then encountered in corresponding waking life. One aim of this work is to test multiple hypotheses stemming from the simulation theories.

Specifically, *social simulation* (Dresler, 2015; Revonsuo et al., 2015a,b; Tuominen et al., 2019) states that dream content will overrepresent social interactions relative to waking life, and this serves to strengthen waking life social perceptions and bonding skills. This construct operates on a natural biological basis for dreaming, such that the social function of dreaming is presumed to increase reproductive fitness, by “rehearsing” anticipated, probable social events. The function of these biased social dream contents is to either aid in re-inclusion in the case of a diminished social life or maintain inclusion in a group (Gardner et al., 2000; Pickett et al., 2004; Gardner et al., 2005; Tuominen et al., 2021).

Research today demonstrates some support for the social predisposition of dreams. There is overall consistency in the appearance of dream characters that coincides with the self’s social realities—characters often appear similarly in dreams as they do in real life (Kahn et al., 2000; Revonsuo et al., 2015b), and a great proportion of dream characters consist of individuals the self is familiar with in waking life (Kahn et al., 2000, 2002; Kahn and Hobson, 2003; Windt, 2018) and a large frequency of interactions in dreams occurs with familiar individuals (Tuominen et al., 2021). Social networks are prevalent in dreams where the self is alone less than 5% of the time, more characters appear in dreams than in waking life (Revonsuo, 2000; Revonsuo et al., 2015b; Han et al., 2016; Domhoff and Schneider, 2018) and dreams have been shown to influence subsequent relational behaviors (Selterman et al., 2013). In addition, psychological well-being, a psychometric measure that has been correlated with social support (Thoits, 1985), has been associated with prosocial dream contents (Pesant and Zadra, 2006). In comparative studies contrasting wake and dream phenomenology, dreams have been found to have greater proportions of social situations than waking life (McNamara et al., 2005; Tuominen et al., 2019). Theory of mind, or “mindreading,” is an evolutionarily advantageous social cognitive function which is a feature retained in dreams that has been hypothesized to aid in social bonding (Kahn and Hobson, 2005; McNamara et al., 2007). In fact, social cognitive neural pathways that support the theory of mind model are highly activated in rapid eye-movement sleep, specifically the activation of the anterior cingulate which functions to regulate emotions, aids in problem solving, and helps to adapt to changing environments (Devinsky et al., 1995; Erwin and Hof, 2001; Vogeley et al., 2001; Franklin and Zyphur, 2005). This coincides with the observation that dream experiences of the self often probe the intentional states of others and is taken literally despite the dream being an unreal experience (Franklin and Zyphur, 2005; Tuominen et al., 2021). Taken together, these findings suggest that social information may be differentially biased to appear in dreams and therefore may have been evolutionarily advantageous.

By contrast, when the self is involved in precarious environments that pose a threat to survival (Revonsuo, 2000), *threat simulation* claims that dream contents should be overall more negative in nature (Revonsuo, 2000), ultimately serving to strengthen waking threat perception skills and threat avoidance behaviors that help the self-cope with the challenging realities of waking life (Revonsuo, 2000; Revonsuo and Valli, 2000, 2008; Revonsuo et al., 2015a). This threat simulation mechanism consists of two parts: (1) *threat recognition simulation* which serves to recognize threats faster over time and (2) *threat avoidance simulation* which serves to implicitly rehearse the appropriate response to that threat (Revonsuo, 2000). Both stages are presumed to lead to increased performance in waking life once a similar event is encountered, because the necessary neural circuit connections to execute those actions have already been primed during sleep (Revonsuo, 2000).

Overall, research has shown mixed support for the threat simulation function of dreams. Some studies have shown that threats that occurred in dreams were unrealistic, and the dream self was unable to successfully evade threats in majority of dreams (Malcolm-Smith and Solms, 2004; Malcom-Smith et al., 2008). Zadra et al. (2006) found mixed support for the threat simulation function of dreams—although most threat simulation propositions were empirically supported in this study, a large proportion of dream threats were also found to be unrealistic. On the contrary, it has been found that threatening events occurred more frequently in dreams than in waking life, with most threats targeted to the dream self, patients with REM sleep behavioral disorder report more aggressive dreams relative to healthy controls, and dreams collected from traumatized children are more negative relative to controls (Revonsuo and Valli, 2000; Fantini et al., 2005; Valli et al., 2005, 2008).

Similar to an immune response, it is hypothesized that the frequency of threatening dream contents may increase during hypervigilant contexts as a means to prepare the self for real-life dangers (Revonsuo, 2000). That is, the threat simulating mechanism is only fully activated in the face of valid ecological cues like disease, illness, and predation risks (Revonsuo, 2000). Thus, the SARS-CoV-2 virus (COVID-19 henceforth), which would otherwise be difficult to experimentally mimic, can be considered a unique opportunity in which threat simulation predictions can be tested under natural conditions within a mutually exclusive hypothesis design. This presented an opportunity to investigate dream contents during the pandemic.

For this reason, explorations of evolutionary theories on dream functions have become especially relevant during the COVID-19 pandemic (Iorio et al., 2020; Wang et al., 2021). The self is often threatened in dreams and aggression is particularly salient in rapid eye-movement sleep dreams (Uguccioni et al., 2013) and it has been found that most dream reports have at least one threatening element (Revonsuo, 2000; Dale et al., 2016). A study by Mota et al. (2020) suggests that a large percentage of dreams collected during COVID-19 make specific references to viral risk, illness, and mental suffering. In a dreams study conducted by Parrello et al. (2021), it was found that individuals that were affected by the virus reported more longer and negatively themed dreams, with higher instances of anger, sadness and anxiety. Put together, these findings suggests that the pandemic, which under the threat simulation framework would qualify as an evolutionarily relevant survival risk (Revonsuo, 2000), could potentially influence dream content. In this vein, the scientific study of the function of dreams is theoretically robust, yet the need for research that critically evaluates threat and social simulation constructs by testing them is urgently needed (Dresler, 2015; Revonsuo et al., 2015b; Domhoff and Schneider, 2018). This is the primary aim of our study.

Currently, there are four hypotheses that stem from the simulation theories of dream function, of which will be the object of exploration here. The *social bias hypothesis* states that dream states overrepresent positive social situations relative to waking life (Tuominen et al., 2019). We predicted that positive social situations in dreams are greater than in waking life. Second, if dreams are more social than waking life, then we should expect to see more individuals in dreams relative to waking states. We predicted that dreams should feature more characters than in corresponding waking life.

A mutually exclusive alternative to the social bias hypothesis that will be considered here is the *threat bias hypothesis*, the idea that dream contents will be threatening relative to waking life, as opposed to featuring positive dream contents (Revonsuo and Valli, 2000; Tuominen et al., 2019). A unique opportunity to test these hypotheses, the COVID-19 pandemic provides natural environmental cues of risk that would otherwise be difficult to experimentally mimic. Under this hypothesis, we predicted that dreams should be characterized by more threatening and negative contents relative to waking life (Revonsuo and Valli, 2000; Tuominen et al., 2019).

The *strengthening hypothesis* states that in order for dreams to maintain real-life social inclusion, dreams will bias more positive situations toward individuals familiar to the self (Tuominen et al., 2019). We predicted that dream states will demonstrate more interactions with familiar individuals than in waking states. Lastly, the ***compensation hypothesis*** states that when the self’s social life is diminished, social contents in dreams will increase during periods of social seclusion (Tuominen et al., 2019, 2021). This hypothesis is especially relevant to explore during the COVID-19 pandemic, given that social coordination has at the very least been upended and held uncertain. Here, we predicted that dream states predict more social situations than in waking states, and that lower scores on the social support scale (an operationalization of self-perceived social life) significantly predicts more social situations.

## 2. Materials and methods

### 2.1. Description of sample

A sample of 24 University of Toronto students in the Department of Anthropology in Ontario, Canada, were instructed to provide at minimum 10 wake and 10 dream reports sequentially over 30 days (27 September 2021–25 October 2021). Participants included 21 females and three males between the ages of 19–25 years (mean = 21.9 years, SD = 1.5 years). The total number of reports analyzed in this study were 184 wake reports and 168 dream reports (total *n* = 352). Demographic data were collected from participants following the 2016 Canadian Census of Population on generation status, country of origin and ethnic identification categories (Statistics Canada, 2017). Participants were asked “What is [their] generation status to Canada?” and to “Please select [their] country of origin,” both questions are to be answered by separate drop-down menus.

The sample of dream and waking reports were collected during the fourth wave of the COVID-19 pandemic, where the proliferation of COVID-19 variants was of major concern in Ontario, Canada (Public Health Agency of Canada, 2021). During this time, self-rated mental health was below national average (<50%; Statistics Canada, 2021), and 82% of the Canadian population that were eligible for vaccination were fully vaccinated, however restrictions were still imposed in most areas, including mask-wearing, and limiting contacts (Public Health Agency of Canada, 2021).

In addition, we aimed to account for potentially relevant confounders that could impact dream contents underrepresented in previous literature, including sleep quality using the Pittsburgh.

Sleep Quality Index (Buysse, 1989), stress (Cohen et al., 1983), social support (Sherbourne and Stewart, 1991), and media exposures operationalized by the Attention to Health Topics Media Exposures Scale (Sherbourne and Stewart, 1991). To this end, 24 participants from the University of Toronto were recruited to participate in a dreaming study over the course of 30 days. Subjects were instructed to provide wake and dream reports using the Most Recent Dream methodology (Domhoff, 2000) and were then analyzed using a slightly revised version of the Social Content Scale (Revonsuo and Valli, 2000; Tuominen et al., 2019; Wang et al., 2021). Generalized linear mixed effects models following the Poisson distribution were implemented to account for repeated measures while controlling for relevant confounders such as: report contributions per participant, stress, social support, sleep quality, and media exposures (Schredl, 2010).

Participants were prompted the following question for collecting data on ethnic categories: “The 2016 Canadian Census” identifies the following ethnic categories in its Census of the Population. Please indicate how you self-identify. If you are of mixed descent, please indicate this by checking all that apply’ and were provided a list of ethnic categories of which multiple selections can be submitted. Participants were ethnically and nationally diverse (see Supplementary Table 1; Supplementary Figures 1–3 for descriptive data on generation status and ethnicity of the sample). Thereafter, participants were instructed to complete the Pittsburgh Sleep Quality Index as a general measure for sleep quality (Buysse, 1989), the Perceived Stress Scale (Cohen et al., 1983), the MOS Social Support Scale (Sherbourne and Stewart, 1991), and the Attention to Health Topics Media Exposures Scale (Romantan et al., 2008). After the study period, participants were financially compensated. This study was approved by the University of Toronto Review Ethics Board under Protocol #00039768.

### 2.2. Data collection and content analysis methodology

Participants were instructed to submit wake and dream reports between 50 and 250 words at the same time every day. Wake and dream reports were submitted through an online survey that prompts participants to describe how their day went and a recount of their dream experience, noting down any instances of emotional valence, and social interactions with others (Domhoff, 2000; see Supplementary Table 2 for exact prompts used).

Both wake and dream reports were analyzed using a revised version of the Social Content Scale (SCS) proposed by Tuominen et al. (2019). The SCS contains elements that overlap with the Dream Threat Scale (DTS; Wang et al., 2021): the nature of threatening events in the DTS is similar to indicators under the qualities of social situations in the SCS. The DTS, originally proposed by Revonsuo and Valli (2000), is used to detect threatening social content in written reports. In this study, the SCS-revised has been consolidated to include aspects of both the SCS and DTS, by including additional measures for threatening content under qualities of social situations. This revised scale can now detect instances of social content that is either positive, neutral, and negative, including interactions of a threatening nature. This has been conducted similarly in Wang et al. (2021), and overcomes previous limitations associated with using the SCS and DTS separately when testing world-simulation theories (see Supplementary Table 3).

Similar to the SCS, the SCS-revised includes rating measures for: (1) initiating characters, (2) recipient characters, (3) the type of social situation detected, (4) the quality of the social situation, and (5) the tense of the event. Any character (real or fictional) can be coded so long as their presence or perceptions are implied. Here, each category is treated as an independent variable. The rater must first identify in order of ascending number per dream and wake report: the social event, and then the social situation. There can be multiple social situations within a social event, whereby the nature and characters involved in a social situation change. The rater then identifies the five indicators in the detected interaction (for additional instructions see Tuominen et al., 2019). Two raters analyzed the 352 reports using the revised version of the SCS. Interrater agreement rates were calculated for seven categories (social event, social situation, identity of a character, group status of a character, sex of a character, social situation type, and social situation quality) using Cohen’s Kappa Landis and Koch Criteria, where a Kappa value between 0.00–0.20 indicates slight agreement, 0.21–0.40 indicates fair agreement, 0.41–0.60 indicates moderate agreement, 0.61–0.80 indicates substantial agreement, and 0.81–1.00 indicates almost perfect agreement (Landis and Koch, 1977).

### 2.3. Statistical procedure

Where appropriate, paired, and unpaired two-sample tests were used to generate descriptive statistics on reports. To account for individual effects, general linear mixed effects models (GLMM) design under the Poisson family (i.e., counts) using the *lme4* package were introduced for each subcategory of interest (Schredl, 2010). The response variables of interest included subcategories measured from the Social Content Scale (Tuominen et al., 2019). These include positive social situations, negative social situations, characters, familiar individuals, unfamiliar individuals, and social situation counts per individual report. Models were ran on the level of individual reports (*n* = 352). Analyses were preformed using R version 4.1.0 (R Core Team, 2018). For each model, we considered the following fixed effects, with “subjectID” as a random effect to account for subjects’ repeated measures:


$$\begin{array}{l} Model\;1:Positive\;Social\;Situations\;\sim \;report\;state\;\left(wake\right)\\ \;+report\;contributions+perceived\;stress\\ \;+social\;support+media\;exposures\\ \;+PSQI+sex+|subjectID| \end{array}$$

$$\begin{array}{l} Model\;2:Negative\;Social\;Situations\;\sim \;report\;state\;\left(wake\right)\\ \;+report\;contributions+perceived\;stress\\ \;+social\;support+media\;exposures\\ \;+PSQI+sex+|subjectID| \end{array}$$

$$\begin{array}{l} Model\;3:Familiar\;Individuals\;\sim \;report\;state\;\left(wake\right)\\ \;+report\;contributions+perceived\;stress\\ \;+social\;support+media\;exposures\\ \;+PSQI+sex+|subjectID| \end{array}$$

$$\begin{array}{l} Model\;4:Social\;Situations\;\sim \;report\;state\;\left(wake\right)\\ \;+report\;contributions+perceived\;stress\\ \;+social\;support+media\;exposures\\ \;+PSQI+sex+|subjectID| \end{array}$$

$$\begin{array}{l} Model\;5:Characters\;\sim \;report\;state\;\left(wake\right)\\ \;+report\;contributions+perceived\;stress\\ \;+social\;support+media\;exposures\\ \;+PSQI+sex+|subjectID| \end{array}$$

$$\begin{array}{l} Model\;6:Unfamiliar\;Individuals\;\sim \;report\;state\;\left(wake\right)\\ \;+report\;contributions+perceived\;stress\\ \;+social\;support+media\;exposures\\ \;+PSQI+sex+|subjectID| \end{array}$$

## 3. Results

### 3.1. Sample descriptive statistics and interrater reliability

Participants provided on average seven wake reports (SD = 3 wake reports) and seven dream reports (SD = 3 dream reports). A paired Wilcoxon test revealed that the number of wake reports and dream reports provided per participant did not significantly differ (*V* = 90, *p*-value = 0.38). Wake and dream reports were on average 108 words (SD = 46 words) and 121 words (SD = 44 words) in length, and a Wilcoxon test revealed that word lengths between wake and dream states significantly differed (*W =* 18,544, *p*-value < 0.05). A two-sample *t*-test showed that females reported significantly longer wake reports (mean = 110 words, SD = 47 words) than males (mean = 91 words, SD = 33 words) (*t* = 2.06, *df* = 182, *p*-value < 0.05, CI 95% [0.86, 38.43]). This was also the case for dream reports: a two-sample t-test showed that females (mean = 124 words, SD = 46 words) reported significantly longer dream reports than males (mean = 104 words, SD = 26 words) (*t* = 1.98, *df* = 166, *p*-value < 0.05, CI 95% [0.10, 38.4]). For the interrater assessment, Cohen’s Kappa indicated that there was fair to moderate agreement across the seven categories (0.30–0.55; see Supplementary Table 4).

### 3.2. Generalized linear mixed effects model results

Since report contributions across report state and per subject varied, GLMMs were introduced to account for individual effects. These were ran on the level of individual reports under the Poisson family (total sample in this model *n* = 352). Response variables of interest were, in order: (1) positive interactions, (2) negative interactions, (3) familiar individuals that were interacted with by the self, (4) social situation counts, (5) characters, and (6) unfamiliar individuals that were interacted with by the self, per report. The reference categories for report state and sex were, respectively, dreams relative to waking states and females relative to males. Fixed effects such as: report state (dream or wake), sex (female or male), report number, stress, social support, media exposures, and PSQI, and random effects such as subject ID were considered, as a function of each response variable of interest. In addressing the issue of uneven report contributes per participant, all GLMMs indicated that after controlling for relevant confounders, report number was an insignificant predictor to each subcategory of interest (see Table 1 for GLMM results).

**Table 1:** Six GLMMs were introduced as a function of number of (1) counts of positive social situations, (2) counts of negative social situations, (3) counts of familiar individuals and (4) social situation counts, (5) character counts, and (6) counts of unfamiliar individuals per report.

	Β	SE	*z*	*p*	AIC	CI (95%)
GLMM 1: Positive social situations					806.3	
*Report state (wake)*	**0.63**	**0.12**	**4.98**	**<0.001**		**[0.38, 0.88]**
*Report contributions*	0.03	0.02	1.79	0.07		[−0.003, 0.07]
*Perceived stress*	−0.01	0.01	−1.09	0.27		[−0.03, 0.01]
*Social support*	0.004	0.004	0.96	0.33		[−0.004, 0.01]
*Media exposures*	−0.03	0.02	−1.73	0.08		[−0.08, 0.04]
*PSQI*	−0.02	0.03	−0.62	0.53		[−0.08, 0.04]
*Sex (male)*	−0.00	0.19	−0.01	0.99		[−0.38, 0.38]
GLMM 2: Negative social situations					519.4	
*Report state (wake)*	**−1.17**	**0.21**	**−5.44**	**<0.001**		**[−1.59, −0.75]**
*Report contributions*	−0.03	0.03	−1.23	0.22		[−0.10, 0.02]
*Perceived stress*	0.02	0.02	0.93	0.35		[−0.02, 0.06]
*Social support*	0.01	0.007	1.44	0.14		[−0.004, 0.02]
*Media exposures*	−0.04	0.04	−1.02	0.30		[−0.12, 0.04]
*PSQI*	0.02	0.05	0.48	0.62		[−0.07, 0.12]
*Sex (male)*	−0.57	0.37	−1.53	0.12		[−1.30, 0.16]
GLMM 3: Familiar individuals					831.5	
*Report state (wake)*	0.10	0.11	0.91	0.36		[−0.11, 0.32]
*Report contributions*	0.01	0.01	1.18	0.23		[−0.01, 0.04]
*Perceived stress*	0.009	0.01	0.87	0.38		[−0.01, 0.02]
*Social support*	0.003	0.003	0.99	0.32		[−0.003, 0.01]
*Media exposures*	0.02	0.02	1.29	0.19		[−0.01, 0.06]
*PSQI*	−0.02	0.02	−1.08	0.27		[−0.08, 0.02]
*Sex (male)*	−0.02	0.17	−0.16	0.87		[−0.36, 0.31]
*Positive social situations*	**0.40**	**0.05**	**7.28**	**<0.001**		**[0.29, 0.51]**
GLMM 4: Social situations					1061.4	
*Report state (wake)*	−0.11	0.07	−1.43	0.15		[−0.26, 0.04]
*Report contributions*	0.008	0.01	0.70	0.48		[−0.01, 0.03]
*Perceived stress*	−0.01	0.009	−1.43	0.15		[−0.03, 0.005]
*Social support*	0.001	0.003	0.29	0.77		[−0.006, 0.008]
*Media exposures*	−0.02	0.01	−1.27	0.20		[−0.05, 0.01]
*PSQI*	−0.02	0.02	−0.80	0.41		[−0.06, 0.02]
*Sex (male)*	−0.16	0.15	−1.06	0.28		[−0.47, 0.14]
GLMM 5: Characters					994.9	
*Report state (wake)*	**−0.16**	**0.08**	**−1.98**	**<0.05**		**[−0.32, −0.002]**
*Report contributions*	0.01	0.01	0.97	0.04		[−0.01, 0.03]
*Perceived stress*	−0.01	0.008	−1.58	0.32		[−0.03, 0.003]
*Social support*	0.003	0.003	1.07	0.28		[−0.002, 0.009]
*Media exposures*	−0.01	0.01	−1.06	0.28		[−0.04, 0.01]
*PSQI*	−0.002	0.02	−0.09	0.92		[−0.04, 0.04]
*Sex (male)*	−0.15	0.14	−1.07	0.28		[−0.43, 0.12]
GLMM 6: Unfamiliar individuals					711.1	
*Report state (wake)*	**−0.96**	**0.14**	**−6.70**	**<0.001**		**[−1.24, −0.68]**
*Report contributions*	−0.02	0.02	−1.30	0.19		[−0.07, 0.14]
*Perceived stress*	**−0.03**	**0.01**	**−2.10**	**<0.05**		**[−0.06, −0.002]**
*Social support*	−0.0009	0.005	−0.15	0.87		[−0.01, 0.01]
*Media exposures*	−0.05	0.02	−1.78	0.07		[−0.11, 0.005]
*PSQI*	0.02	0.04	0.61	0.53		[−0.05, 0.10]
*Sex (male)*	−0.29	0.26	−1.07	0.28		[−0.81, 0.23]

Displayed here are estimate coefficients (β), standard error (SE), z scores (z), *p* values (p), AIC, and confidence intervals (CI) per fixed effect. Bolded fixed effects are significant. Parentheses indicate reference categories. See Supplementary Figures 3–13 for fixed effects and prediction plots.

Model 1 and Model 5 which both explored the *social bias hypothesis* (see Table 1 for GLMM results) showed that waking states demonstrate more positive social situations than in dreams (*β* = 1.63, *SE* = 0.12, *p* < 0.001, 95% CI [0.38, 0.88]) and that waking states featured fewer characters than in dream states (*β* = −0.16, *SE* = 0.08, *p* < 0.05, 95% CI [−0.32, −0.002]). Model 2, which explored the *threat bias hypothesis*, showed that dream states were more negative in nature relative to waking states even after controlling for relevant confounders (*β* = −1.17, *SE* = 0.21, *p* < 0.001, 95% CI [−1.59, −0.75]). GLMM 3, which explored the *strengthening hypothesis*, shows that prosocial dream contents were significantly associated with familiar individuals (*β* = 0.40, *SE* = 0.05, *p* < 0.01, 95% CI [0.29, 0.51]) however this was impartial to the state of the report. Model 6 revealed that lower stress levels significantly predicted fewer interactions with unfamiliar individuals (*β* = −0.03, *SE* = 0.01, *p* < 0.05, 95% CI [−0.06, −0.002]) and waking states were associated with fewer interactions toward unfamiliar individuals (*β* = −0.96, *SE* = 0.14, *p* < 0.001, 95% CI [−1.24, −0.68]). Model 4, which explored the *compensation hypothesis*, indicated that report state (*β* = −0.11, *SE* = 0.07, *p* = 0.15, 95% CI [−0.26, 0.04]) and perceived social support (*β* = 0.001, *SE* = 0.003, *p* = 0.77, 95% CI [−0.006, 0.008]) were not significantly related to the frequency of social situations the self is engaged.

## 4. Discussion

**Figure 1:**
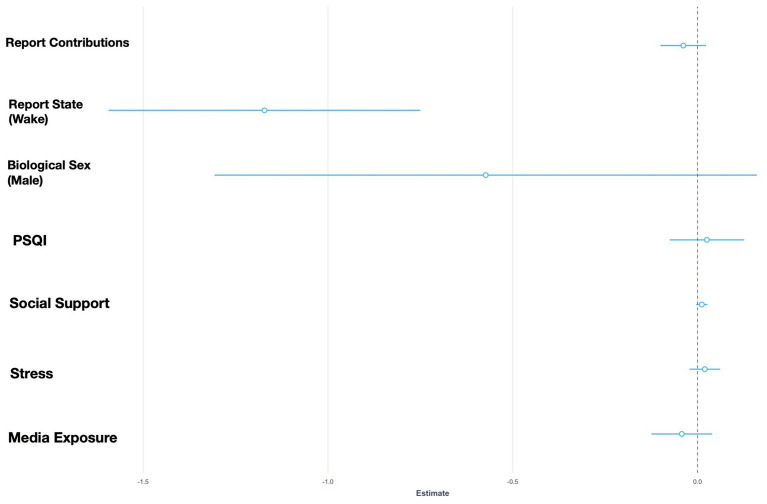
Fixed effects plot for GLMM Model 2 with negative social situations as the response variable.

**Figure 2:**
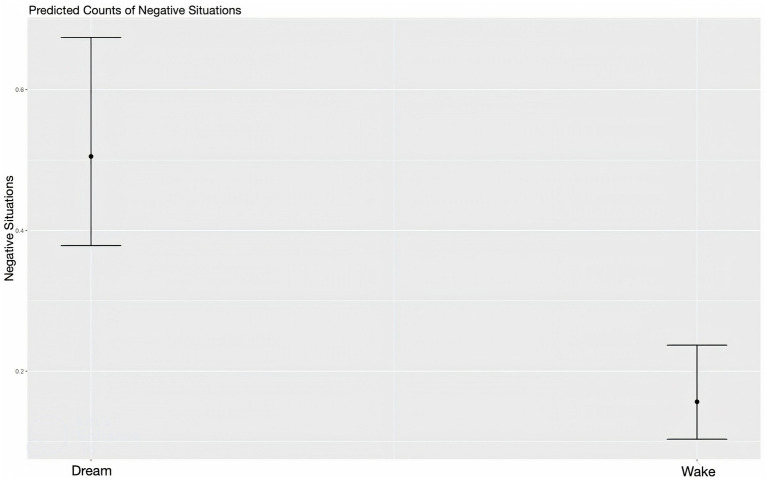
Predicted values (marginal effects) for report state and negative social situations.

The statistical models revealed partial support for the social bias hypothesis—although more characters appeared in dreams than in waking states, the frequency of prosocial situations were not related to dream states. Instead, the sample featured greater frequencies of positive social situations in waking life than in dreams. The model measuring negative social situations, however, did demonstrate support for the alternative threat bias hypothesis—dreams were characterized by more negative social situations and interactions toward unfamiliar individuals than in waking life (see Figures 1, 2). Interestingly, it was found that lower levels of stress predicted fewer interactions toward unfamiliar individuals. The model that explored the strengthening hypothesis revealed partial support—prosocial interactions were mainly with familiar individuals however this is not particular to wake or dream states. Lastly, the compensation hypothesis was not supported in this study.

The finding that dream contents were negative is congruent with a substantial amount of dream literature published during the COVID-19 pandemic. Monterrosa-Castro et al. (2020) found that healthcare providers in Colombia often had COVID-19 related nightmares associated with Generalized Anxiety Disorder. Pesonen et al. (2020) conducted a computational network analysis study on dream reports collected during the lockdown period and found that majority of dreams were pandemic specific (>50%) and referred to themes such as failures in social distancing, avoiding crowded areas, coronavirus contagion, dystopia, and apocalyptic themes. These distressing dream themes were accentuated in participants with higher stress levels (Pesonen et al., 2020). A dreaming study conducted on American participants found that individuals who reported being most affected by the pandemic were more likely to remember their dreams often, of which were often pandemic-themed and were largely negatively toned (Schredl and Bulkeley, 2020). This study also interestingly revealed that restrictions posed on social interactions resulted in more negatively toned dreams (Schredl and Bulkeley, 2020).

A study conducted by MacKay and DeCicco (2020) found that COVID-19 dreams were significantly related to increases in viral imagery, animal imagery, and location changes. When comparing COVID-19 dreams to pre-lockdown dreams in Italy, it was found that COVID-19 dreams were higher in emotional load and bizarreness (Gorgoni et al., 2021). Although it may make sense to claim that pandemic dream studies show support for threat or social simulation theories (threat simulation in particular), accounts for waking life experiences are missing here to be able to directly contrast dreams to experiences encountered during the day—which would be necessary evidence to gather to be able to test threat or social simulation hypotheses or to be able to make claims related to these hypotheses in general. These studies have generally shown support for the incorporation continuity hypothesis, where general concerns are consolidated and reflected in dreams.

An interesting finding reported by several studies during COVID-19 found biological sex differences in dream content—females typically have more negative dreams during the pandemic relative to males (Barrett, 2020; Schredl and Bulkeley, 2020; Gorgoni et al., 2021; Kilius et al., 2021). This finding was not corroborated in this study—this is most likely due to an uneven sample sex balance. Alternatively, an explanation to account for this is that the sample of dreams in this study were collected during a period (October 2021) where lockdown restrictions were rather lax relative to restrictions earlier on during the pandemic, and vaccine uptake had substantially increased around this time in Canada. This may have perhaps decreased associated day-time stressors for females which then affected respective dream contents. In general, to better delineate COVID-19’s influence on dream content, future studies should aim to measure changes in dream content relative to waking life, alongside associated psychometric measures (i.e., stress, psychological well-being), as societies eventually progress from the pandemic into a new normal.

The role of unfamiliar individuals in dreams is unclear (see Supplementary Figures 11, 12). Our results show that the self is more likely to interact with unfamiliar people in dreams than in waking life. In this study, unfamiliar individuals were coded as characters that were only known *via* their occupational roles, were unspecified, or were strangers. This finding coincided with the threat bias hypothesis under the threat simulation framework, as people generally tend to avoid interacting with strangers (Sandstorm and Boothby, 2020). Alongside this finding, contingency analysis (see Table 2) revealed that negative and threatening thematic contents occurred more frequently in dreams than in waking life, including themes such as: physical violence, verbal aggression, forcing behaviors, abandonment, escapes and pursuits, failure, and disease and illness. It is possible that unfamiliar individuals were preferred agents in which the self “practiced” relevant threat perception skills and avoidance behaviors toward.

**Table 2:** Subcategories of social situations in wake (*n* = 184) and dream (*n* = 168) reports.

	Wake (*n*)	Wake (%)	Dream (*n*)	Dream (%)	*p*-value
**Positive**					
Physical affection	0	0%	2	0.59%	
Verbal affection	0	0%	0	0%	
Consentful sexual	0	0%	0	0%	
Altruistic behavior	70	21.15%	33	9.68%	
Approach cues	104	31.42%	33	9.68%	
Request for support	19	5.74%	14	4.11%	
Mediating behavior	0	0	9	2.64%	
*Total positive interactions*	193/331	58.31%	91/341	26.68%	<0.05
**Negative**					
Physical violence	0	0%	10	2.93%	
Verbal aggression	4	1.21%	9	2.64%	
Forcing	0	0%	6	1.76%	
Unconsentful sexual	0	0%	0	0%	
Avoidance behavior	3	0.91%	0	0%	
Abandonment	0	0%	1	0.29%	
*Total negative interactions*	7/331	2.12%	20/341	5.86%	0.01
**Threatening**					
Escapes and pursuits	2	0.60%	28	8.21%	
Accidents and misfortunes	3	0.91%	2	0.59%	
Failures	0	0%	2	0.59%	
Catastrophes	0	0%	0	0%	
Disease and illness	0	0%	1	0.29%	
*Total threats*	5/331	1.51%	33/341	9.67%	<0.05
**Neutral**					
*Neutral interactions*	110/331	33.23%	82/341	24.05%	0.01
**Perceptions**					
*Perception only*	7/331	2.12%	84/341	24.63%	<0.05
**Perceptions with emotional reactions**					
Positive	3	0.91%	8	2.35%	
Negative	6	1.81%	23	6.74%	
*Total reactions*	9/331	2.72%	31/341	9.09%	<0.05

Fisher’s Exact tests were used to assess significance per subcategory on a presence or absence basis. See Figure 1 for visualized proportions.

This is substantiated by the finding that in Model 6, lower levels of waking day stress predicted fewer interactions with unfamiliar individuals, and that perceptions occurred more frequently in dreams than in waking life (see Tables 1, 2; Supplementary Figure 13). This overlaps with literature that states that perception cognition by way of mental imagery (i.e., the formation of representations in the mind) is associated with higher goal achievement, since internal representations of actions generally involve the activation of the same brain areas when compared to the motor execution of said actions occurring (Knauper et al., 2009; Cumming and Williams, 2012). Higher stress levels may predict more negatively toned interactions with unfamiliar individuals in dreams, which may function to help the self “practice and prepare” in adequately dealing with waking day dilemmas.

### 4.1. Limitations

Some limitations are present in this study design. Most importantly, although we hypothesized that dreams collected during the COVID-19 pandemic will be more negative relative to waking life, a pre-pandemic sample is missing in our analysis here that would have otherwise been introduced as a control to effectively measure the potential influence of the pandemic on dream content. As a result, we cannot say for certain that the threat bias in dreams in this sample is exclusively due to the pandemic. We suggest that a longitudinal dream content study will better delineate the effects of the pandemic on dream phenomenology. Secondly, the sample used in the study were from a small unrepresentative group (undergraduate Anthropology students). The conclusions reached in this study can benefit from a larger, more representative sample, and we encourage a replication of our methods with a larger sample as a promising direction. Thirdly, the method of wake recall may have encouraged biased recalls of particularly emotionally charged events during the day that are most likely to be remembered by the participant. Although this is mitigated for as best as possible by the required word count of the report (Domhoff and Schneider, 1998), other report collection methods such as the Experience Sampling Method (ESM) as conducted in Tuominen et al. (2019) and Sterpenich et al. (2020) would possibly encourage less biased recalls.

Interrater reliability measures were low with the SCS; this can be further remedied by either including further agreement discussions between raters or further refining the SCS’s coding instruction units. Additionally, the presence of any psychopathologies was not screened for in participants, which has been correlated to more negative dream contents (Armitage et al., 1995; Levin and Basile, 2003; Knudson, 2006; Wittmann et al., 2010; Soffer-Dudek et al., 2011; Soffer-Dudek and Sadeh, 2012; Mota et al., 2014; Banu et al., 2017; Soffer-Dudek, 2017; Sikka et al., 2018; Gupta, 2020; Martin et al., 2020; Solomonova et al., 2021). In addition, future work could provide additional social psychometric measures which can be introduced to the study that align with the social simulation framework. This could include interpersonal attachment styles (Leary et al., 1995, 1998; McNamara et al., 2001; Selterman and Drigotas, 2009; Tuominen et al., 2021) which has been shown to correlate with dream contents. Along this line, collecting objective sleep measures on participants to correlate to specific dream contents may substantiate further evolutionary claims regarding dream functions (Snyder, 1966; Tuominen et al., 2021).

Critical to the study of social and threat simulation hypotheses is the claim regarding increased cognitive performance following exposure to social or threatening dream contents, which is missing here. Future work should execute an experimental design that explores if exposure to certain dream contents relates to an increase in cognitive performance in tasks that are appropriate proxies for threat and social perceptions.

## 5. Conclusion

In summary, our main findings are that dreams are more negative and feature more unfamiliar individuals in contrast to waking life. From an evolutionary perspective, the functions of dreams are an exciting and underexplored domain. Further studies in this area could have immediate applications in therapeutic interventions, with the power to inform diagnostic markers of psychopathology (Armitage et al., 1995; Mota et al., 2014; Martin et al., 2020). Dream reports are invaluable sources that allows for insight into structural thinking—a diagnostic marker and reliable predictor of clinical disorders including schizophrenia and clinical depression (Armitage et al., 1995; Mota et al., 2014; Sikka et al., 2018; Martin et al., 2020). Broadly construed, these findings imply that dreams may have evolved as a kind of functional heuristic that aids in day-time decision-making with regards to threat perception. This function may have been evolutionarily advantageous, such as increasing the likelihood of survival rates in Paleolithic ancestral environments. The function of dreaming has been a topic of interest since the dawn of recorded history, yet empirical research has only recently been able to test functional hypotheses. Further research in this area may contribute to our understanding of how a fundamental mechanism, sleep and dreaming, drives sociality and other fitness related functions. The socioemotional processes related to dreaming and REM sleep, that may facilitate for adaptive social behaviors upon awakening (Thakkar and Datta, 2001; McNamara et al., 2004)—may have been a keystone to human adaptation throughout human evolution. More broadly, further oneirology research will demystify what many consider a mystical experience and can ultimately deepen our scientific understanding of consciousness.

## Data availability statement

The raw data supporting the conclusions of this article will be made available by the authors, without undue reservation.

## Ethics statement

This study was approved by the University of Toronto Review Ethics Board under Protocol #00039768. The patients/participants provided their written informed consent to participate in this study.

## Author contributions

NA and DS conceived of the presented idea. NA preformed data collection, data analysis, and manuscript writing. DS provided feedback and helped with interpretation of results. All authors contributed to the article and approved the submitted version.

## Funding

This research was funded by the University of Toronto Mississauga Teaching Development and Innovation Grant and the Connaught New Researcher Award.

## Conflict of interest

The authors declare that the research was conducted in the absence of any commercial or financial relationships that could be construed as a potential conflict of interest.

## Publisher’s note

All claims expressed in this article are solely those of the authors and do not necessarily represent those of their affiliated organizations, or those of the publisher, the editors and the reviewers. Any product that may be evaluated in this article, or claim that may be made by its manufacturer, is not guaranteed or endorsed by the publisher.
