# Supplementary material for: Dreaming during the COVID-19 pandemic: Support for the threat simulation function of dreams
Source: Front Psychol. 2023 Feb 6;14:1124772. doi: 10.3389/fpsyg.2023.1124772 (PMC9939895; doi:10.3389/fpsyg.2023.1124772)
Supplement: Supplementary file 1 [file Data_Sheet_1.docx]

Supplementary Material

# Supplementary Figures and Tables

## Supplementary Figures


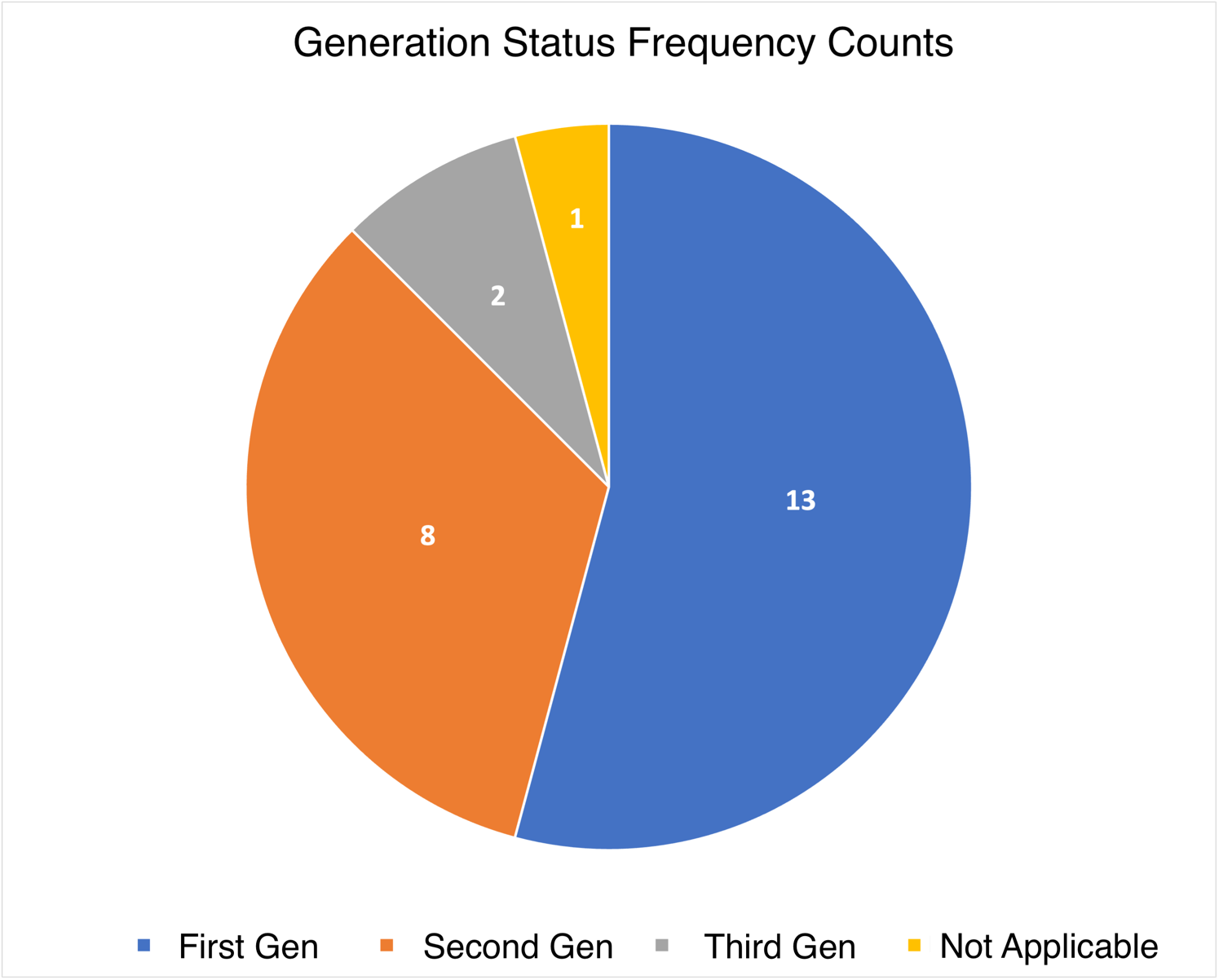


**Supplementary Figure 1.** Visual representation of generation status counts of participants.


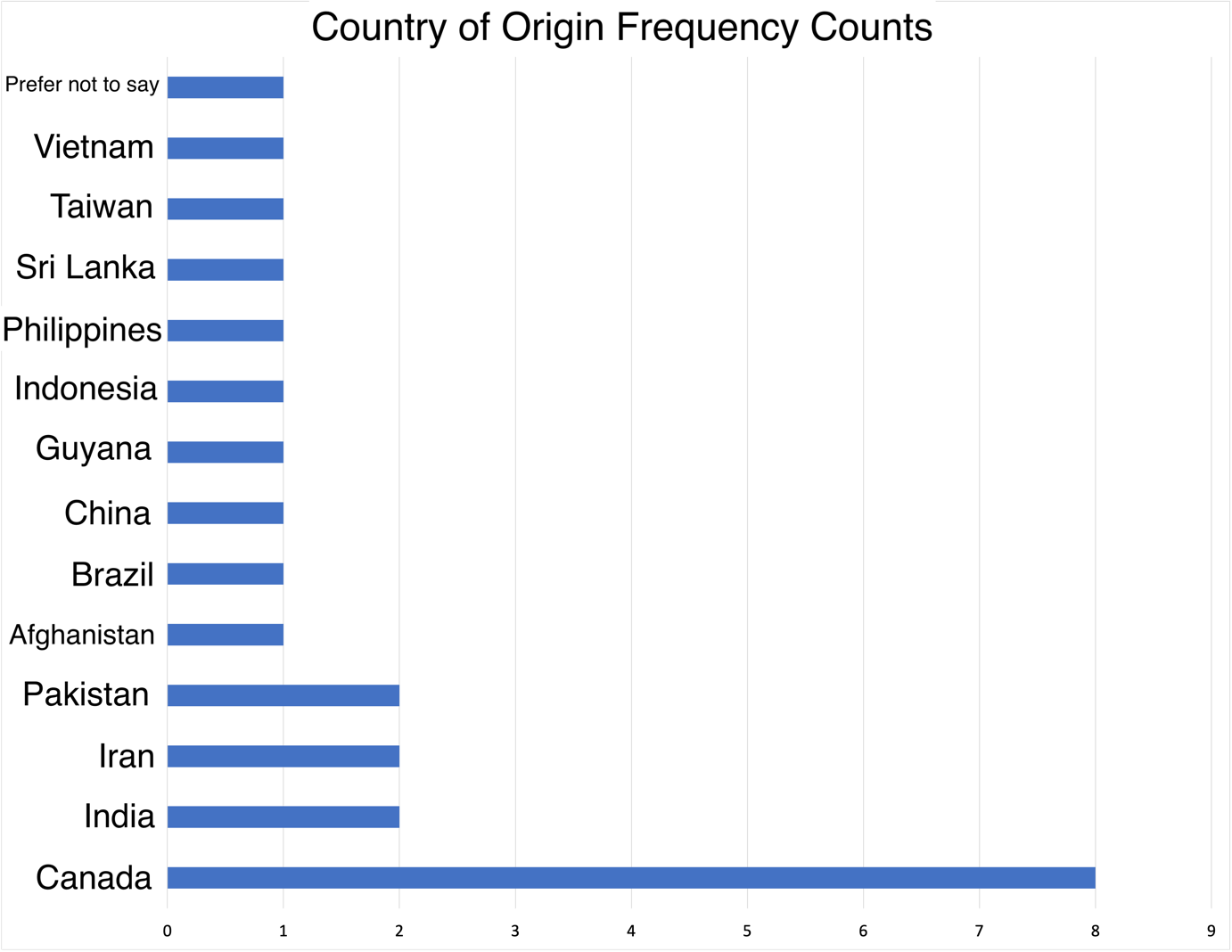


**Supplementary Figure 2.** Visual representation of country of origin counts of participants.


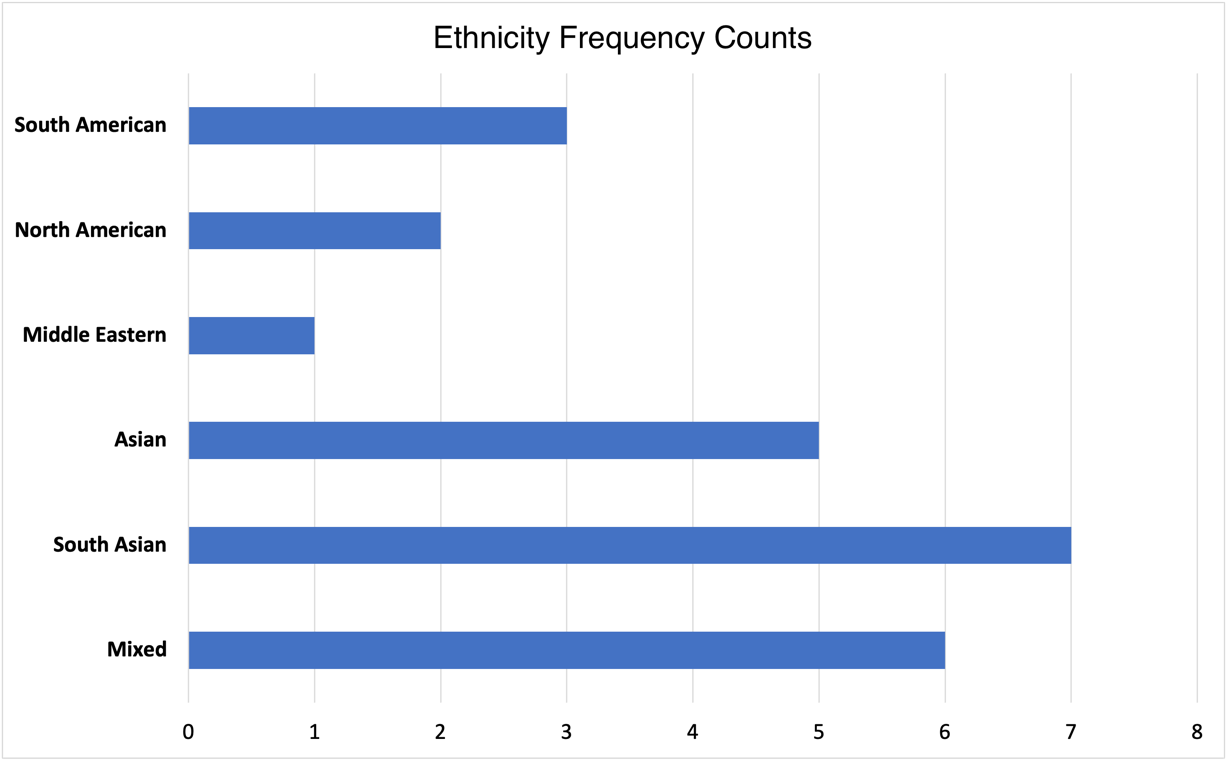


**Supplementary Figure 3.** Visual representation of country of origin counts of participants.


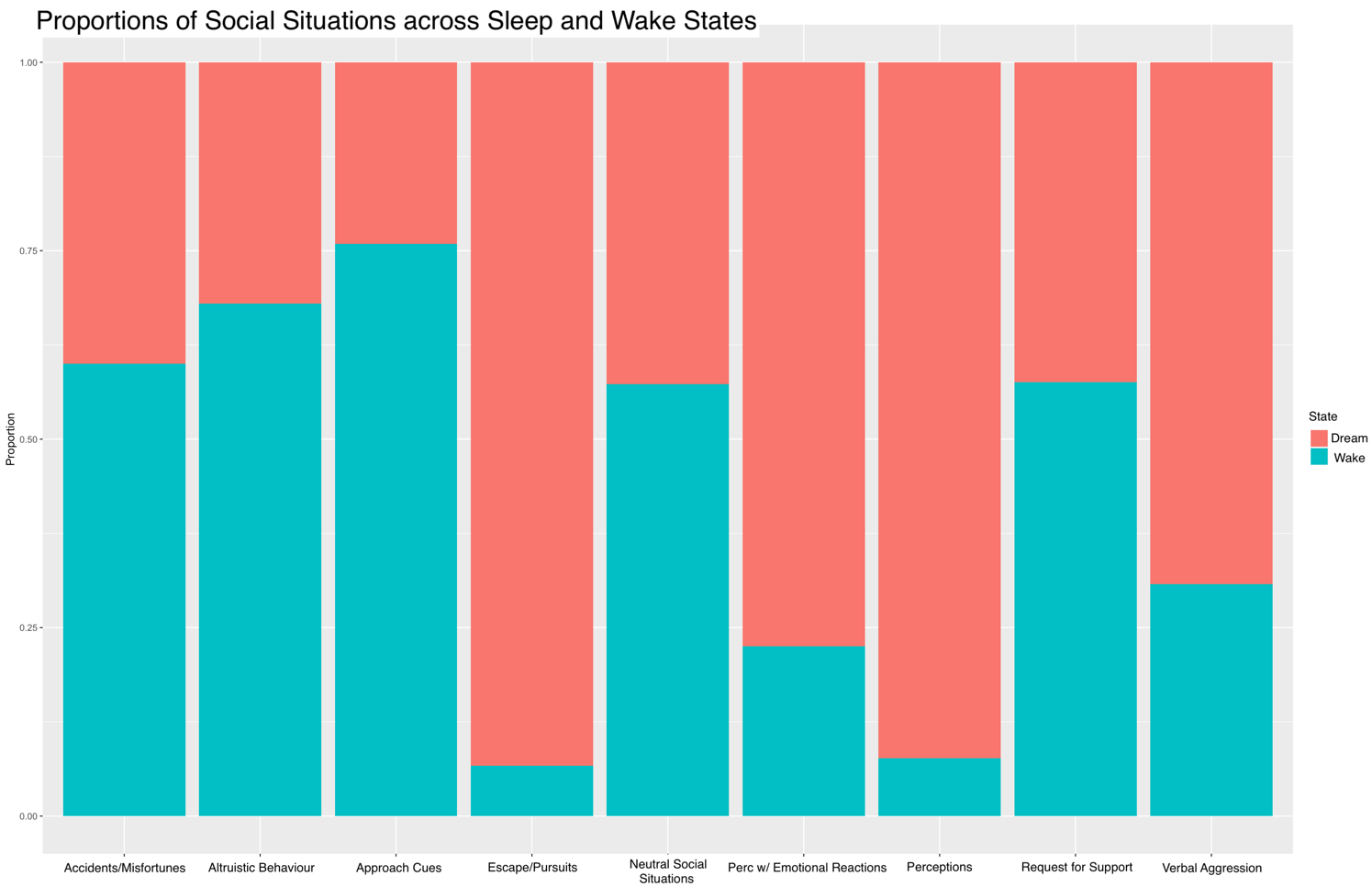


**Supplementary Figure 4.** Visualized proportions of quality of social situations in dream (pink) and wake (blue) states. Subcategories with zero counts in both dream and wake states were removed for visual purposes (catastrophes, unconsentful sexual, and verbal affection). Categories with frequencies in only one state (ie wake or dream) were removed for visual purposes (abandonment, avoidance behaviours, disease/illness, failures, forcing, mediating behaviours, physical affection, physical violence).


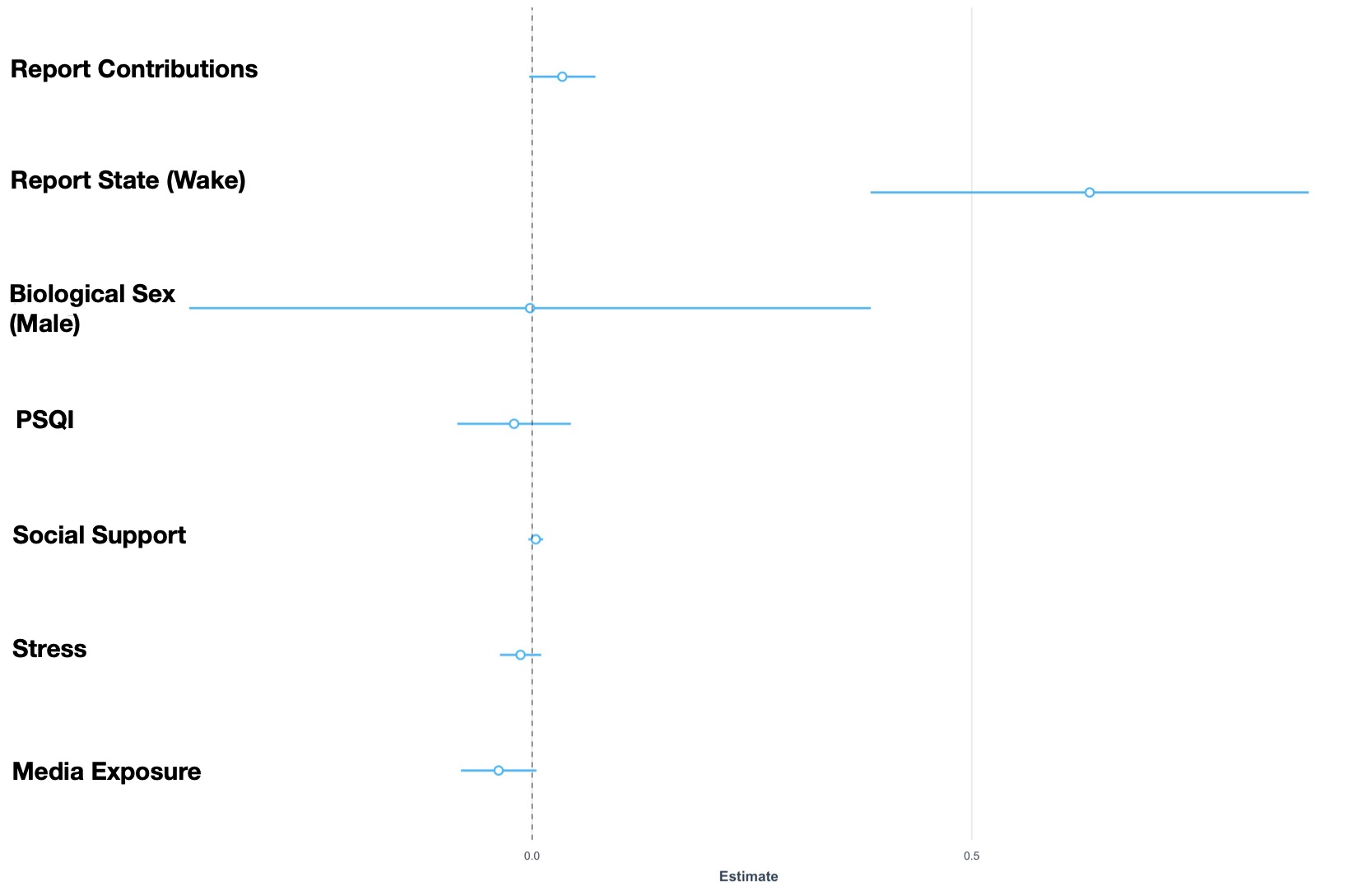


**Supplementary Figure 5:** Fixed effects plot for GLMM Model 1 with positive social situations as the response variable.


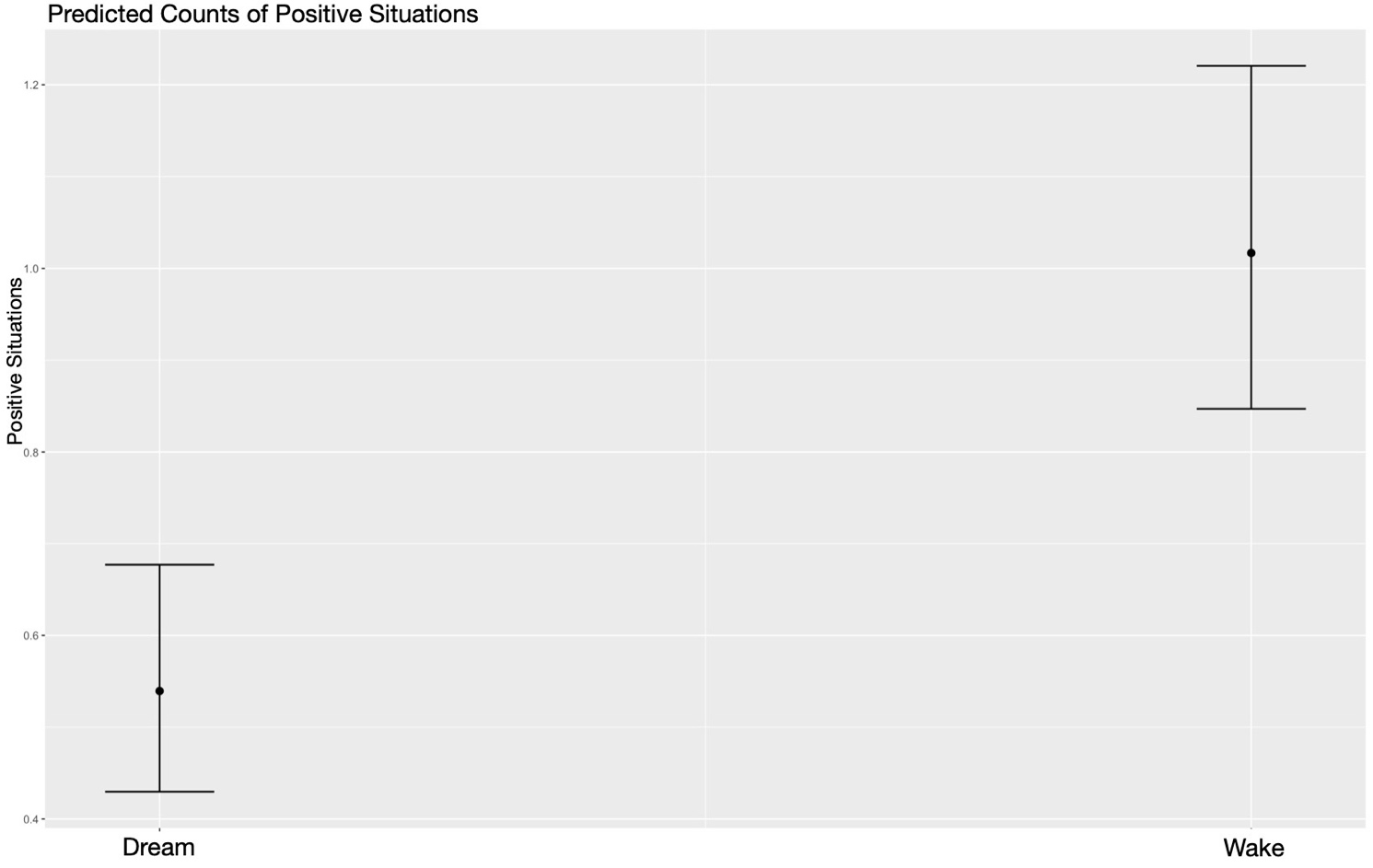


**Supplementary Figure 6:** Predicted values (marginal effects) for report state and positive social situations.


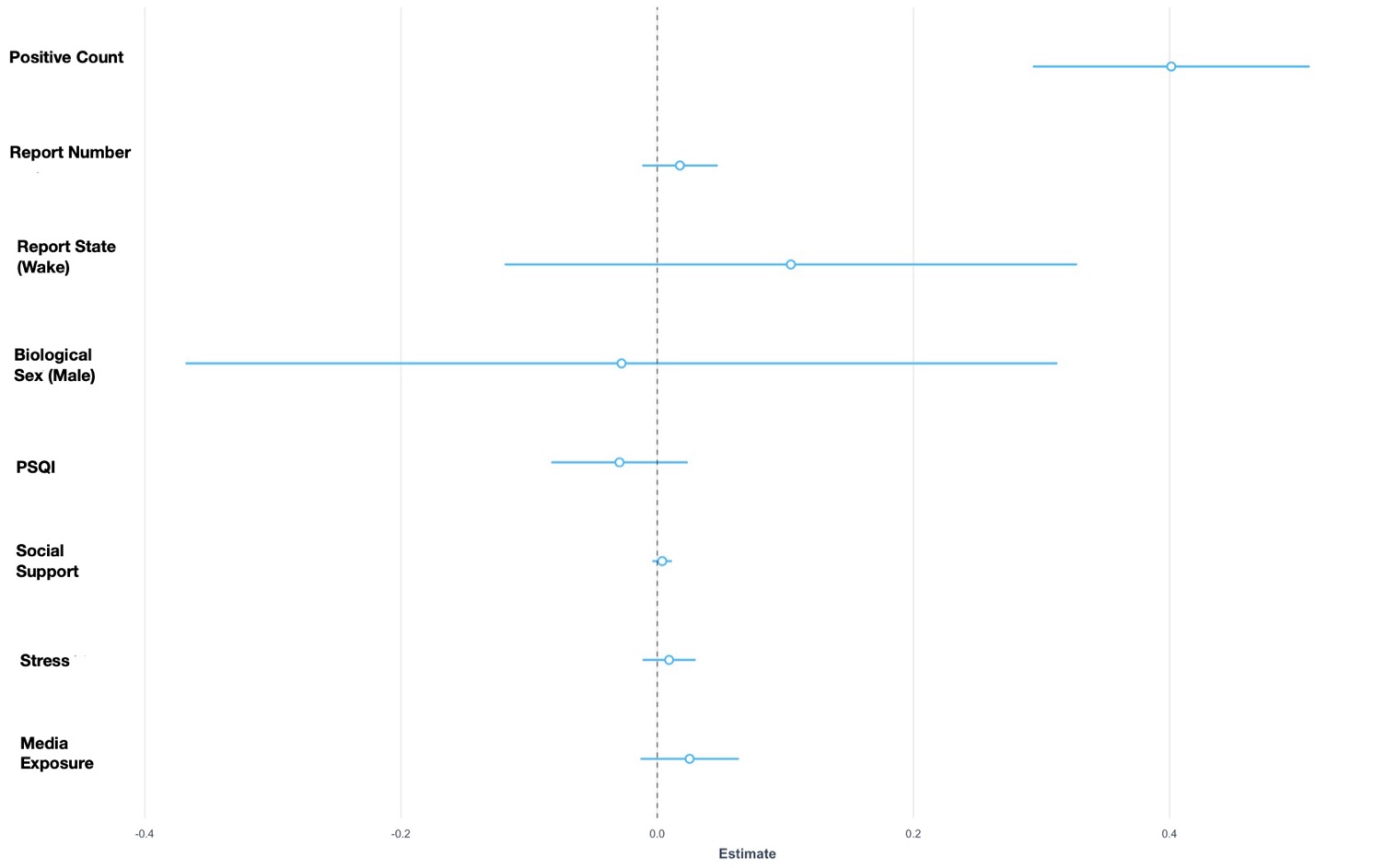
**Supplementary Figure 7:** Fixed effects plot for GLMM Model 3 with familiar individuals as the response variable.


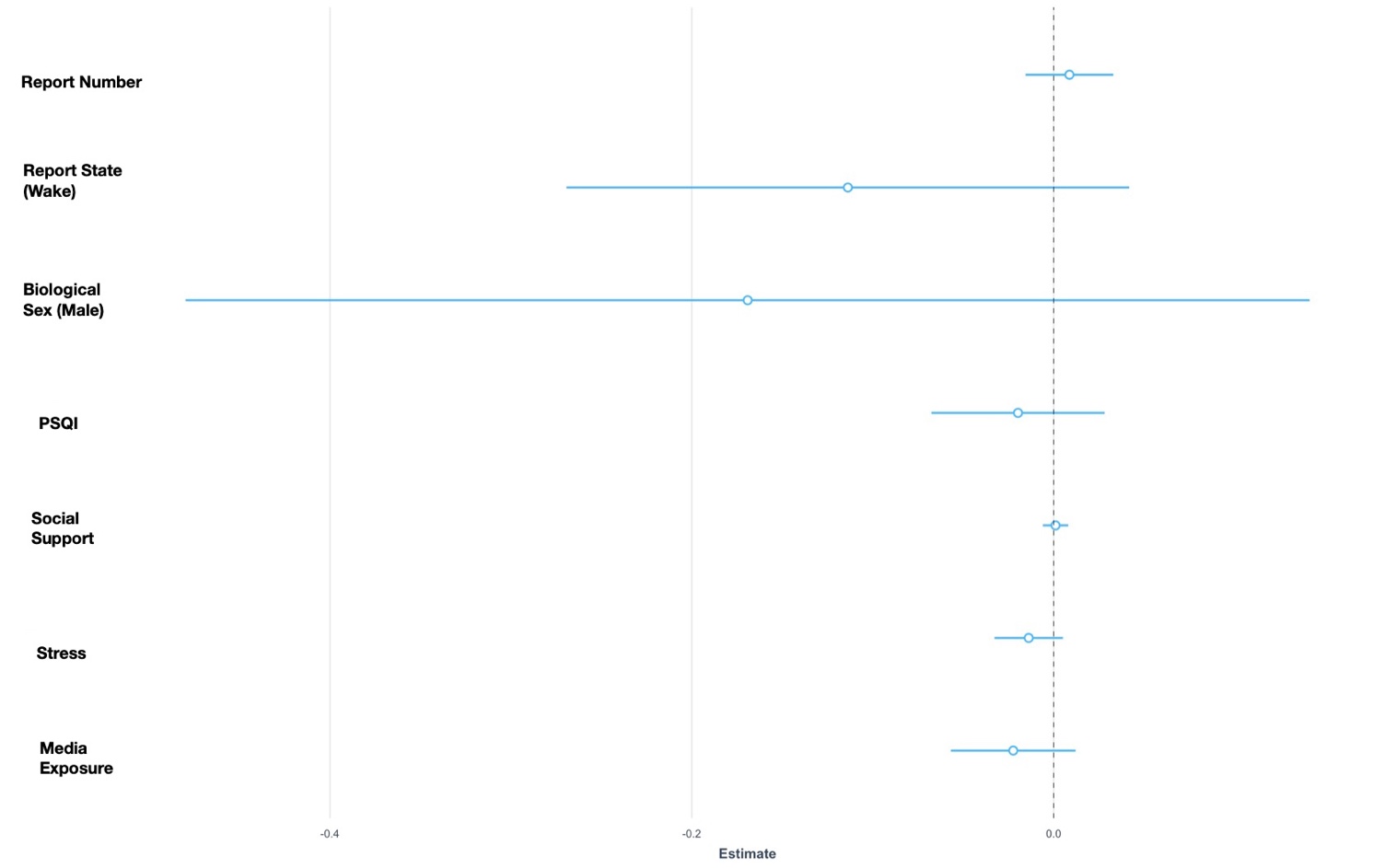


**Supplementary Figure 8:** Fixed effects plot for GLMM Model 4 with social situations as the response variable.


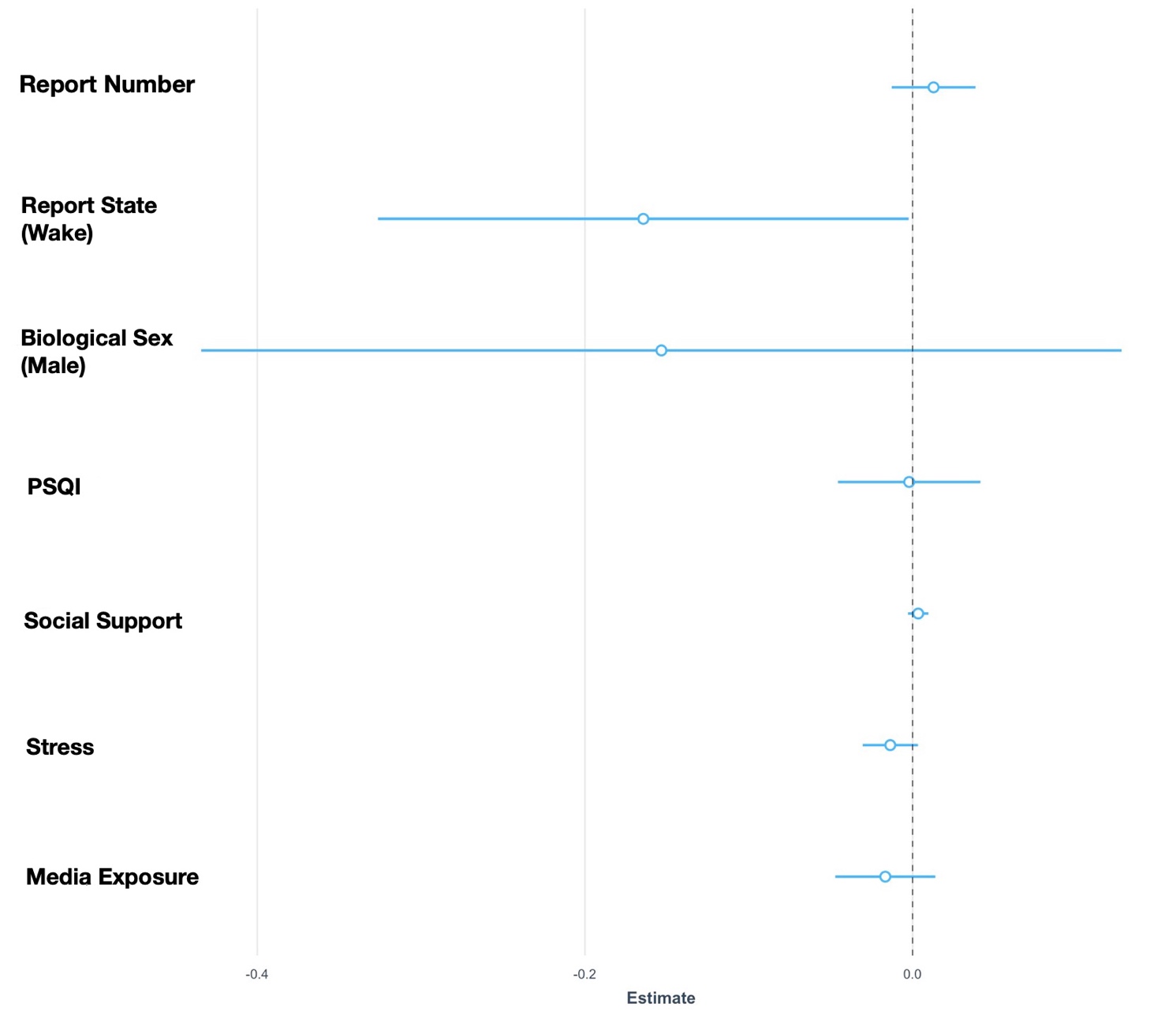


**Supplementary Figure 9:** Fixed effects plot for GLMM Model 5 with characters as the response variable.


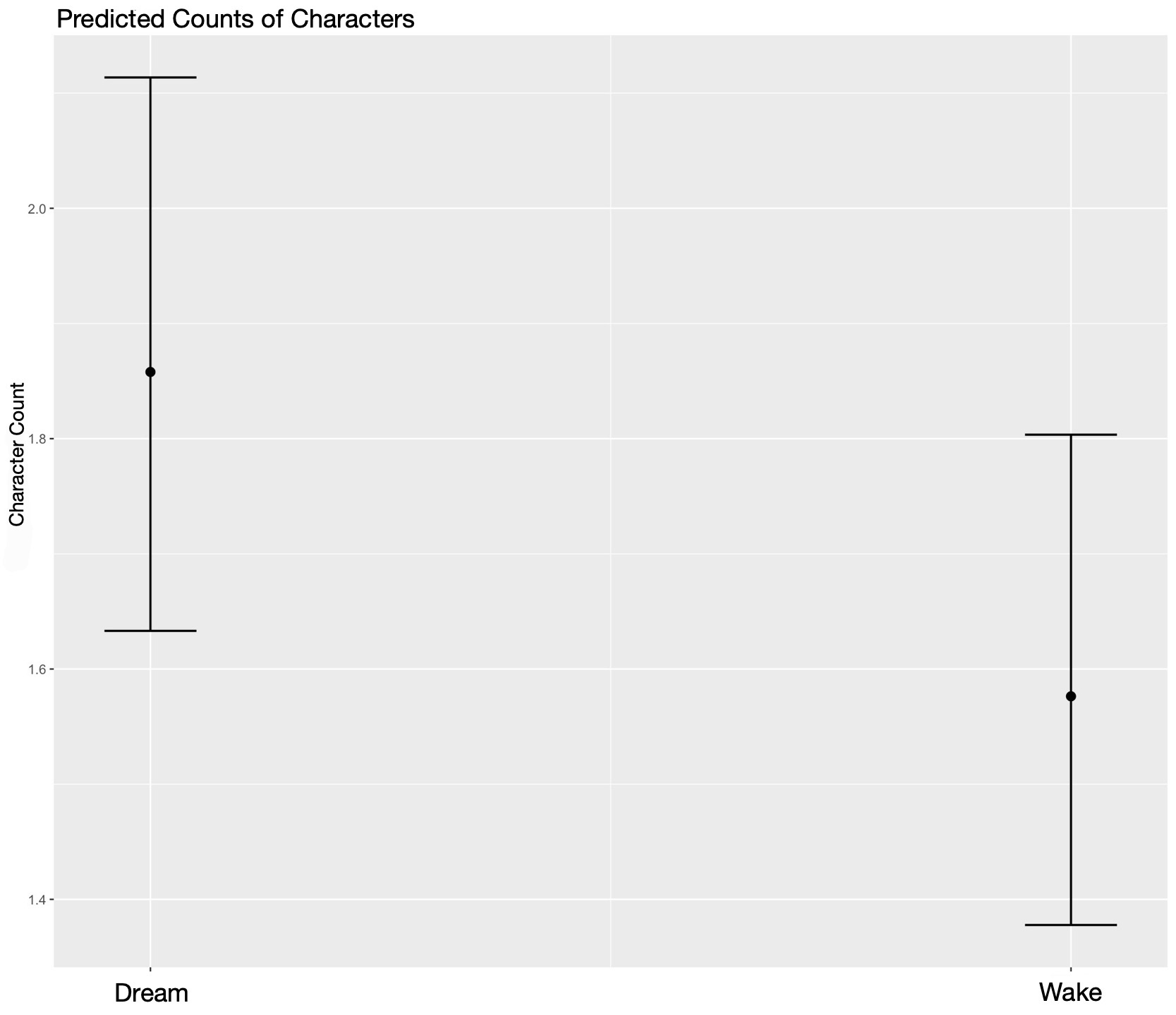


**Supplementary Figure 10:** Predicted values (marginal effects) for report state and characters.


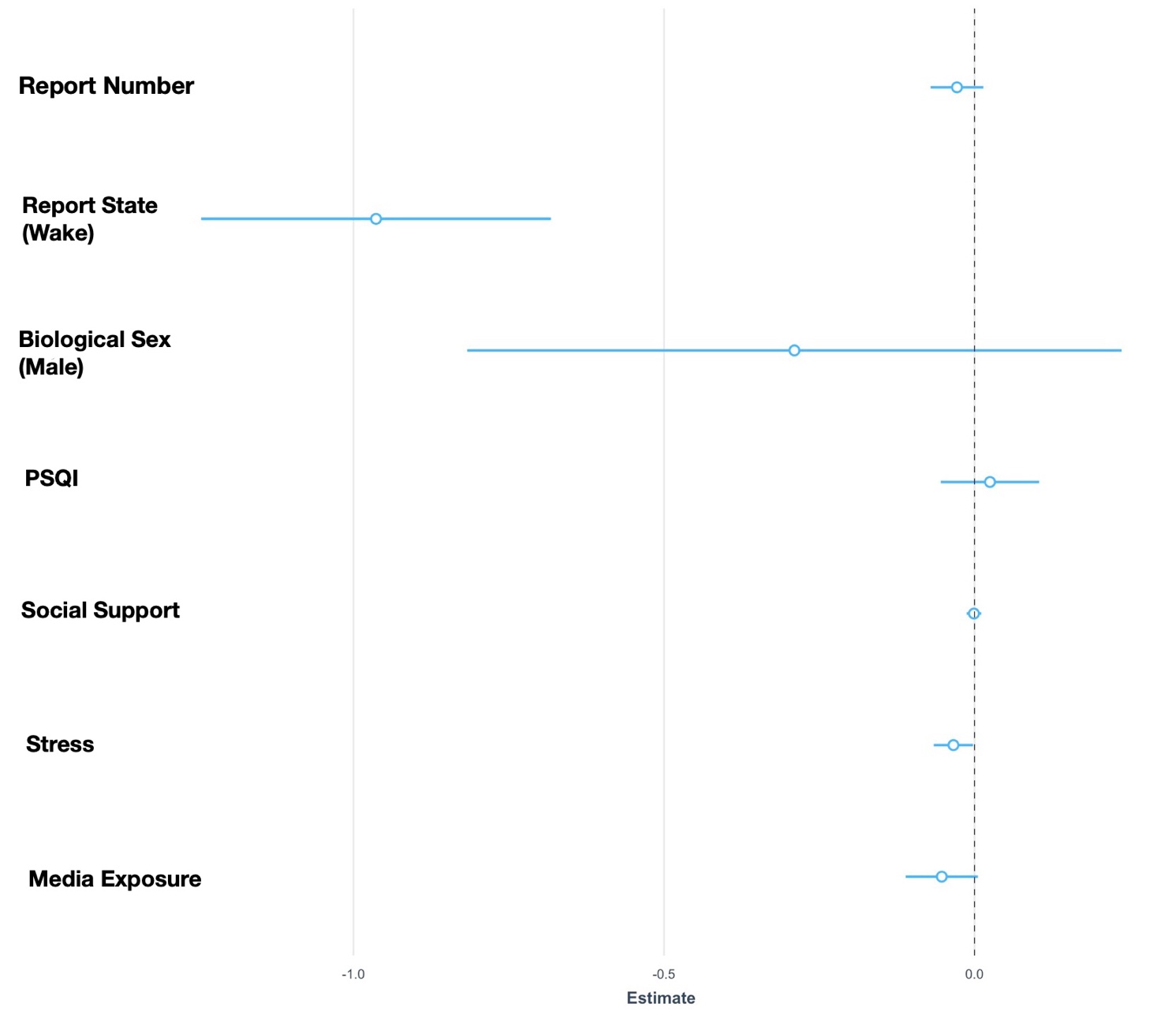


**Supplementary Figure 11:** Fixed effects plot for GLMM Model 6 with unfamiliar individuals as the response variable.


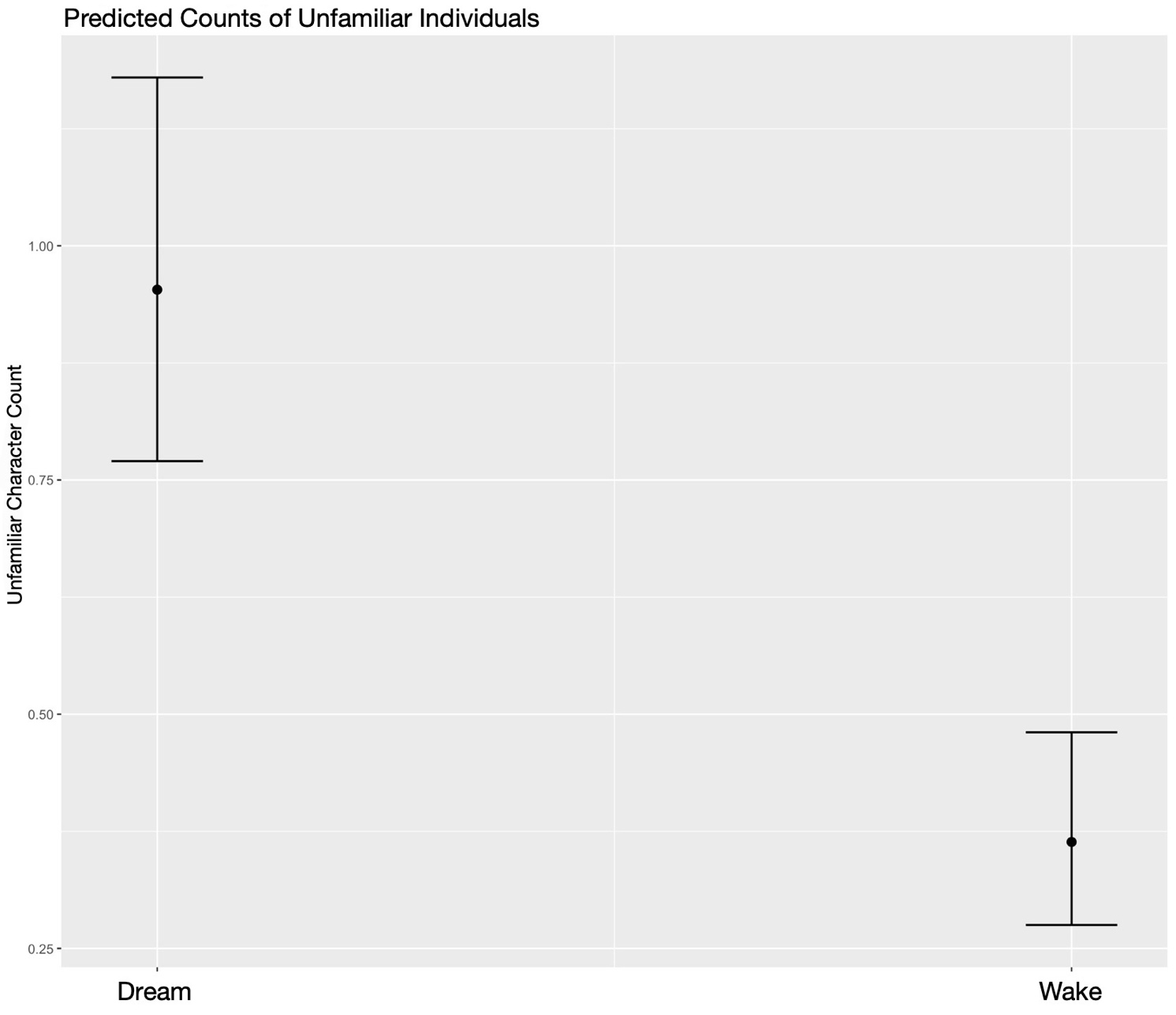


**Supplementary Figure 12:** Predicted values (marginal effects) for report state and unfamiliar individuals.

**
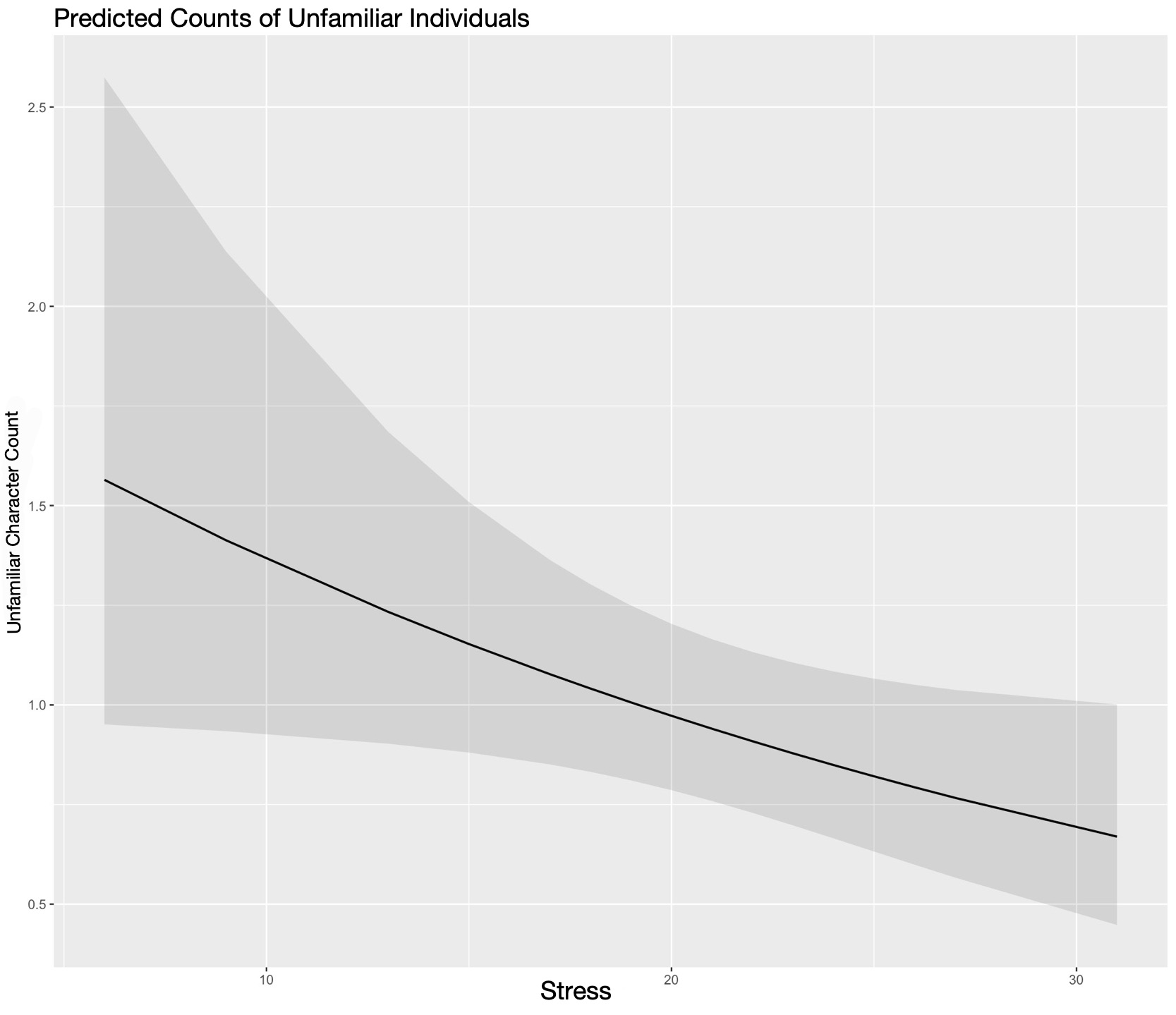

Supplementary Figure 13:** Predicted values for stress and unfamiliar individuals.

## Supplementary Tables

**Supplementary Table 1:** Demonstrating frequencies of country of origins and ethnic identifications of the sample.

| Country Of Origin | Frequency |
| --- | --- |
| *Canada* | 8/24 (33%) |
| *India* | 2/24 (8.33%) |
| *Iran* | 2/24 (8.33%) |
| *Pakistan* | 2/24 (8.33%) |
| *Afghanistan* | 1/24 (8.33%) |
| *Brazil* | 1/24 (4.16%) |
| *China* | 1/24 (4.16%) |
| *Guyana* | 1/24 (4.16%) |
| *Indonesia* | 1/24 (4.16%) |
| *Philippines* | 1/24 (4.16%) |
| *Sri Lanka* | 1/24 (4.16%) |
| *Taiwan* | 1/24 (4.16%) |
| *Vietnam* | 1/24 (4.16%) |
| *Prefer not to say* | 1/24 (4.16%) |
| Ethnic Identification | **Frequency** |
| *South American* | 3/24 (12.5%) |
| *North American* | 2/24 (8.33%) |
| *Middle Eastern* | 1/24 (4.16%) |
| *Asian* | 5/24 (20.83%) |
| *South Asian* | 7/24 (29.16%) |
| *Multiple Ethnicities* | 6/24 (25%) |

**Supplementary Table 2**: Survey prompts used for wake and dream reports.

| **Wake Report Prompt** | Describe how your day went. Your report should contain, whenever possible: significant events that occurred during your day that may have been pleasant or unpleasant, challenges or achievements encountered during the day, your general mood throughout the day, and any places you have visited. |
| --- | --- |
| **Dream Report Prompt** | Describe the dream exactly and as fully as you remember it. Your report should contain, whenever possible: a description of the setting of the dream, whether it was familiar to you or not; a description of the people, their age, sex, and relationship to you; and any animals that appeared in the dream. If possible, describe your feelings during the dream and whether it was pleasant or unpleasant. Be sure to tell exactly what happened during the dream to you and the other characters. |

**Supplementary Table 3**: Revised Social Content Scale (Tuominen et al., 2019; Wang et al., 2021; Revonsuo & Valli, 2000).


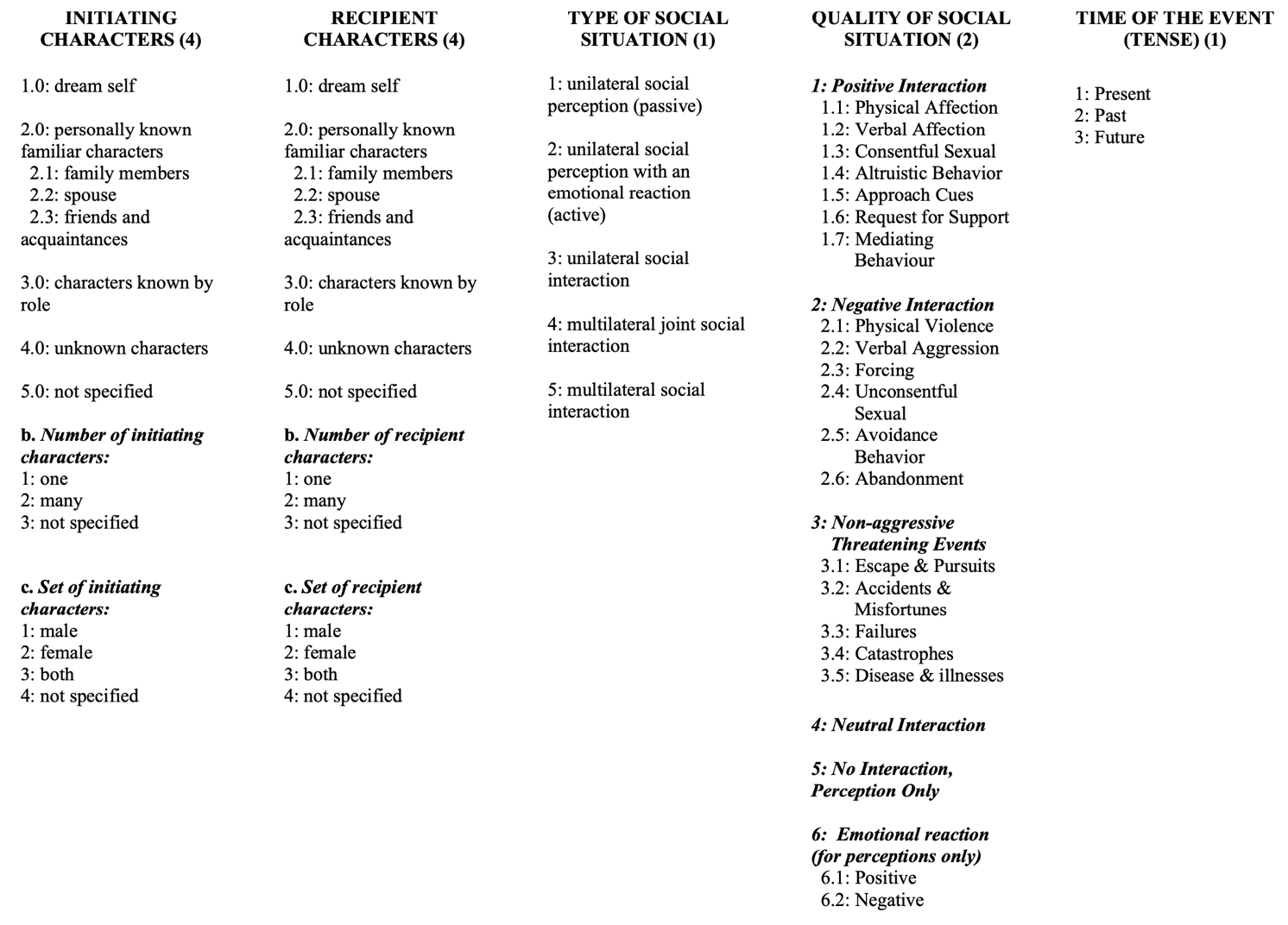


**Supplementary Table 4**: Interrater Reliability using Landis and Koch (1977) Criteria.

| **Category** | **Percentage of Agreement** | **Cohen’s Kappa** | **Standard Error** | **95% CI** | **Agreement** |
| --- | --- | --- | --- | --- | --- |
| Social Event | 69.58% | 0.3 | 0.045 | [0.214, 0.390] | Fair |
| Social Situation | 62.54% | 0.33 | 0.041 | [0.253, 0.412] | Fair |
| Character: Identity | 67.87% | 0.55 | 0.019 | [0.510, 0.583] | Moderate |
| Character: Group Status | 71.25% | 0.43 | 0.023 | [0.380, 0.470] | Moderate |
| Character: Set | 67% | 0.52 | 0.02 | [0.481, 0.559] | Moderate |
| Social Situation Type | 75.16% | 0.45 | 0.037 | [0.377, 0.520] | Moderate |
| Social Situation Quality | 62.29% | 0.43 | 0.031 | [0.372, 0.494] | Moderate |
